# COGNITIVE IMPAIRMENT, DEMENTIA, AND FALLS IN RACIALLY/ETHNICALLY DIVERSE OLDER AMERICANS

**DOI:** 10.1093/geroni/igae098.2583

**Published:** 2024-12-31

**Authors:** Ayse Malatyali, Ladda Thiamwong, Tom Cidav, Rui Xie, Lisa Wiese

**Affiliations:** University of Central Florida, Orlando, Florida, United States; University of Central Florida, Orlando, Florida, United States; U.S. Department of Veterans Affairs (VA), Philadelphia, Pennsylvania, United States; University of Central Florida, Orlando, Florida, United States; Florida Atlantic University, Boca Raton, Florida, United States

## Abstract

Older adults with cognitive impairment (CI) and dementia are prone to falls as they have at least twice as much risk than their cognitively normal peers. The relationship between falls and cognitive decline is bidirectional. However, evidence regarding the effects of falls on CI and dementia is limited. This study describes the association of falls, repeated falls, and related risk factors with CI and dementia among older Americans. This cross-sectional study used datasets from the 2020 interviews of the Health and Retirement Study. The sample included 7,663 participants (age>64). Participants who had fallen in the last two years were 15% more likely to have CI and 34% more likely to have dementia. Increased number of falls was significantly associated with CI (OR=1.03) and dementia (OR=1.04). Having depressive symptoms was also associated with CI (OR=1.64) and dementia (OR=1.99). Participants living in rural areas were 17% more likely to have CI and 53% more likely to have dementia than those living in urban areas. Furthermore, living alone increased the odds of having CI (OR: 1.22) and dementia (OR=1.15). We also observed a significant association of ethnicity and race with cognitive stages: Hispanic participants were 1.7 times more likely, and Black participants were 2.4 times more likely to have CI. The likelihood of having dementia was also higher in Hispanics (OR: 2.36) and Blacks (OR= 5.21) compared to non-Hispanic Whites. Falls prevention interventions should be tailored across cognitive stages in older adults with the consideration of ethnic, racial, and environmental diversities.

